# Clinical Neuropathology image 3-2017: CNS involvement in systemic amyloidosis restricted to the choroid plexus

**DOI:** 10.5414/NP301034

**Published:** 2017-04-24

**Authors:** Ellen Gelpi, Martin Susani, Robert Wiebringhaus, Andreas Aschauer, Andreas Kammerlander, Mirjam I. Lutz, Johannes A. Hainfellner

**Affiliations:** 1 Institute of Neurology,; 2 Department of Pathology,; 3 Department of Medicine II, Medical University of Vienna, Austria, and; 4 Neurological Tissue Bank of the Biobanc Hospital Clinic-IDIBAPS, Barcelona, Spain

**Keywords:** systemic amyloidosis, CNS involvement, plexus choroideus, circumventricular organs

## Abstract

No Abstract available.

We present the neuropathological findings in a 75-year-old man who had the clinical diagnosis of amyloidosis restricted to the heart, which was confirmed by biopsy. The patient died of cardiac insufficiency in the context of arrhythmia. General autopsy revealed amyloid deposits in the heart and additionally in the lung, kidney, thyroid gland, esophagus, pancreas, liver, spleen, periumbilical fat tissue, and rectum. 

In the brain, prominent amyloid deposits were restricted to the vessel walls of the choroid plexus (Figure 1A). There were no deposits in the meninges, CNS parenchyma, or the nerve roots of brainstem. Amyloid deposits were intensely congophilic (Figure 1B, C), birefringent under polarized light, and thioflavin-positive (Figure 1D, arrow). Amyloid deposits were immunoreactive for α- and κ-light chain (Figure 1E), but negative for transthyretin (Figure 1F), amlyoid A, βA4-amyloid, and β2-microglobulin. 

In generalized amyloidoses, amyloid deposits in the CNS have been found in regions were the blood brain barrier is insufficient. This is the case in the choroid plexus, infundibulum, pineal gland, area postrema (representing circumventricular organs), ganglion Gasseri, and dura mater [1, 2], and suggests a hematogenic pattern of spread [3]. Other regions of the brain, such as leptomeninges and brain parenchyma, are devoid of these amyloid deposits, in contrast to what is observed in classical βA4-amyloidosis such as Alzheimer’s disease. 

## Conflict of interest 

The authors report no conflict of interest. 

**Figure 1.**
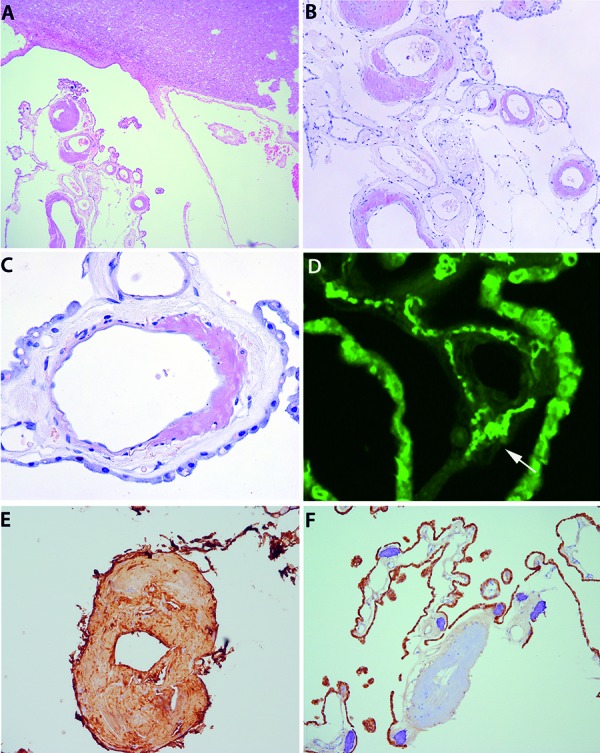
A: Hematoxylin-eosin staining of CNS tissue with adjacent fragments of choroid plexus (lower left). There is striking excentric thickening of the vessel walls of the choroid plexus. B, C, D: The thickened vessel walls contain abundant amorphous deposits showing congophilia (Congo red stain; B ×100; C ×400), and are stained with thioflavin (D; arrow, bright green signal; ×200), corresponding to amyloid. E, F: Immunohistochemistry for α- and κ-light chain (E: κ-light chain ×200) shows immunoreactivity of amyloid deposits in the vessel wall, while immunohistochemistry for transthyretin does not stain those deposits (note the positive staining of the choroid plexus epithelium; ×100; the dark blue structures represent calcifications of the plexus choroideus).
